# Aberrant Right Subclavian Artery Causing Dampening of the Right Radial Arterial Cannula Tracing Following Transesophageal Echocardiography Probe Insertion

**DOI:** 10.14740/jmc5322

**Published:** 2026-06-03

**Authors:** Osama Elazzouny, Joseph D. Tobias, Ibrahim Abdullah

**Affiliations:** a Department of Anesthesiology and Pain Medicine, Nationwide Children’s Hospital, Columbus, OH, USA; b Department of Anesthesiology and Pain Medicine, The Ohio State University College of Medicine, Columbus, OH, USA; c Department of Cardiothoracic and Vascular Surgery, Montefiore Medical Center, Bronx, NY, USA

**Keywords:** Congenital heart disease, Vascular ring, Aberrant right subclavian artery

## Abstract

An aberrant right subclavian artery (ARSA) is the most common congenital anomaly of a left aortic arch. Given the anatomical course of the ARSA, it generally remains asymptomatic throughout life without hemodynamic or respiratory effects. We report the intraoperative diagnosis of ASRA during surgery for congenital heart disease when there was dampening of the waveform from the right radial arterial cannula during transesophageal echocardiography (TEE) probe placement. The subsequent identification of ARSA highlights the importance of considering vascular anomalies in cases of unexplained intraoperative hemodynamic monitoring abnormalities during and after TEE probe insertion.

## Introduction

An aberrant right subclavian artery (ARSA) is the most common congenital anomaly of a left aortic arch and generally remains asymptomatic throughout life [1]. It may occur as an isolated anomaly or in association with more significant congenital heart disease (CHD). The origin of the aberrant artery is generally distal to the take-off of the left subclavian artery from the aortic arch and then crosses the midline in the posterior mediastinum to supply the right upper extremity [1, 2]. In the majority of cases, it crosses behind the esophagus and results in airway or esophageal compression with stridor or dysphagia [3]. We report the unusual presentation and subsequent diagnosis of an ARSA during surgery for CHD in a 6-month-old child with trisomy 21 and a moderate-sized ventricular septal defect (VSD). Unexpected dampening of the waveform from the right radial arterial cannula was noted during transesophageal echocardiography (TEE) probe placement. The subsequent identification of ARSA highlights the importance of considering vascular anomalies in cases of unexplained intraoperative hemodynamic monitoring abnormalities during and after TEE probe insertion.

## Case Report

A 6-month-old, 6.36-kg girl with a history of trisomy 21, moderate VSD, and a small patent ductus arteriosus (PDA) was scheduled for VSD repair and PDA ligation. She was born full-term with an uncomplicated birth history. As she had remained asymptomatic, she presented for elective repair at 6 months of age per our usual practice to prevent future complications including embolism risk, progressive pulmonary hypertension, or congestive heart failure. A preoperative transthoracic echocardiogram (TTE) revealed a moderate membranous VSD, partially covered by aneurysmal tricuspid valve tissue, with a small-to-moderate left-to-right interventricular shunt. Additional findings included a small PDA, a patent foramen ovale (PFO), and trivial tricuspid regurgitation, with no significant mitral regurgitation. Notably, no vascular abnormalities were reported in the preoperative TTE. The patient was otherwise healthy with an unremarkable physical exam associated with the phenotypic features of trisomy 21. The induction of anesthesia and endotracheal intubation were uneventful. The right radial artery was cannulated with a 24-gauge angio-catheter under ultrasound guidance and exchanged for a 2.5-Fr, 2.5-cm catheter using the Seldinger technique with brisk blood return and a normal waveform. A 4-Fr, 5-cm double-lumen central venous catheter was placed in the right internal jugular vein without complications. After TEE probe placement, the right radial arterial waveform became intermittently dampened but then normalized. This finding correlated with TEE probe movement. Troubleshooting confirmed that the arterial catheter remained in place, with no air bubbles present, and there was smooth and brisk blood return. The transducer was replaced; however, the waveform irregularities persisted. As the chest was opened, the surgeon placed a 24-gauge angio-catheter in the first branch of the aorta for continuous monitoring, which displayed a normal waveform. The patient was then placed on cardiopulmonary bypass (CPB). The PDA was ligated, the VSD was closed using a Gore-Tex patch, and the PFO was closed primarily with a Prolene suture. Upon rewarming, the patient regained a normal sinus rhythm and was successfully weaned from CPB. A postoperative TEE showed good biventricular function with no residual septal defect. During the case, the right radial arterial line began functioning more reliably, allowing for removal of the arterial line which was in the first branch of the aorta. Upon chest closure, the right radial arterial line waveform was lost again. This posed a significant surgical dilemma because pulses were palpable while the heart was not visible. A TEE was reperformed which showed good function. It was at this point that we suspected a possible ARSA. Although we wanted to keep the TEE probe in place to continue to monitor the heart with no discernible arterial line tracing, we elected to remove the TEE probe to confirm our suspicion. Upon removing the TEE probe, the right radial arterial waveform returned to normal. This finding suggested the possible presence of an ARSA in the setting of a left aortic arch, which was subsequently confirmed upon re-evaluating the preoperative TTE images. The patient was transported to the pediatric cardiovascular care unit in stable condition. The remainder of the postoperative course was unremarkable. As the patient was asymptomatic without respiratory or hemodynamic symptomatology, no surgical reintervention had been required. Continued outpatient follow-up up to 7 months has demonstrated no residual VSD, no effusions, and good weight gain.

This review was conducted in compliance with the ethical standards of the responsible institution on human subjects as well as with the Helsinki Declaration.

## Discussion

**Figure 1:**
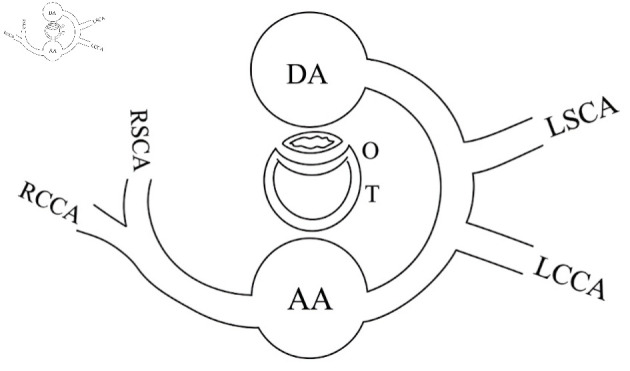
Schematic drawing of a normal left-sided aortic arch and its branches. AA: ascending aorta; LCCA: left common carotid artery; LSCA: left subclavian artery; DA: descending aorta; RSCA: right subclavian artery; RCCA: right common carotid artery; T: trachea; O: esophagus.

**Figure 2:**
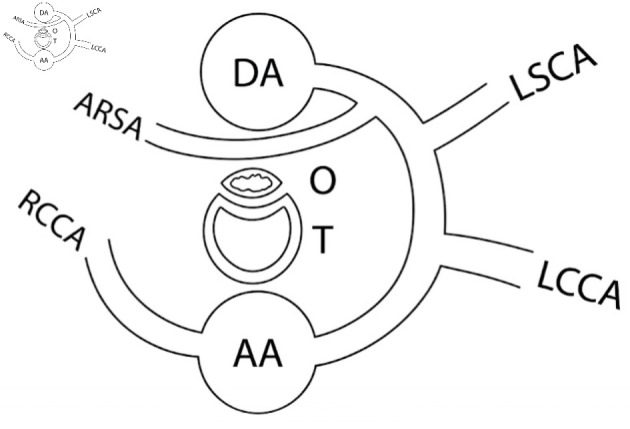
Schematic drawing of a left-sided aortic arch and its branches with an aberrant right subclavian artery (ARSA) arising as the last branch from the distal aortic arch. AA: ascending aorta; LCCA: left common carotid artery; LSCA: left subclavian artery; ARSA: aberrant right subclavian artery; DA: descending aorta; RCCA: right common carotid artery; T: trachea; O: esophagus.

Vascular anomalies are rare congenital anomalies of the aortic arch system. The aortic arch develops during early embryogenesis from a series of six paired aortic arches that arise within the pharyngeal arches, connecting the ventral aorta to the dorsal aorta. Through a complex process of selective growth and regression, the paired aortic arches develop and remodel to form the mature aortic arch and its major branches, including the carotid, subclavian, and pulmonary arteries. Disruptions in this tightly regulated process can lead to congenital anomalies of the aortic arch such as ARSA. Normally, the proximal portion of the right subclavian artery develops from the right fourth aortic arch, whereas the distal portion is derived from the right dorsal aorta and the right seventh intersegmental artery. In ARSA, abnormal regression of the right fourth aortic arch results in failure of formation of the proximal right subclavian artery. Consequently, the right subclavian artery originates from the right seventh intersegmental artery arising from the dorsal aorta and typically takes a retroesophageal course (80% of the cases) to reach the right upper extremity [1] (Figs 1, 2).

Vascular rings encircle the trachea and esophagus. The most common vascular ring is a double aortic arch followed by a right aortic arch with an aberrant left subclavian artery. A left aortic arch with an ARSA is not considered to be a complete vascular ring unless the ligamentum arteriosum is on the right side, which is extremely rare. Complete vascular rings can cause symptoms due to circumferential compression of the trachea and esophagus, including stridor, wheezing, recurrent respiratory infections, feeding difficulties, dysphagia, and choking in infancy. A left aortic arch with an ARSA, though not a complete vascular ring, can cause dysphagia (especially to solids), chronic cough, or mild respiratory symptoms, and some patients may remain asymptomatic [4]. The dysphagia is often termed “dysphagia lusoria” or the “trick of nature”, given that it is not a complete vascular ring.

Evaluation of vascular anomalies begins with a chest radiograph, which may suggest the side of the aortic arch and show tracheal compression. A barium swallow can demonstrate characteristic esophageal indentations. Computed tomography (CT) angiography or magnetic resonance (MR) angiography is the diagnostic gold standard, providing detailed vascular anatomy and essential information for surgical planning. In patients presenting with chronic cough, bronchoscopy performed during cough evaluation may reveal a pulsatile vascular structure compressing the tracheal wall. As our patient was asymptomatic, no preoperative CT or magnetic resonance imaging (MRI) was performed. Echocardiography is recommended in all patients with vascular anomalies, as 12% of vascular anomalies are associated with congenital cardiac lesions, with a VSD being the most common associated cardiac lesion [5]. Scala et al reported the presence of ARSA in 23% of fetuses with Down syndrome undergoing first and second trimester prenatal ultrasonography and recommended its use as a prenatal ultrasound marker for Down syndrome [6]. In this case, the ARSA was asymptomatic, making it more likely to be overlooked on preoperative echocardiography. This highlights the importance of a thorough review of echocardiographic imaging, particularly in patients with a history of Down syndrome. Given that our patient remains asymptomatic, no additional imaging or intervention is planned at this point. He will continue to undergo routine well-child checks with his pediatrician, and additional intervention or imaging may be indicated should he become symptomatic.

Generally, TEE complications in infants are related to the compression of surrounding structures, including the trachea, which can cause difficulty with ventilation or displacement of the endotracheal tube. The probe may also compress adjacent vascular structures such as the descending aorta, causing damping of the femoral arterial line waveform, or, as in this case, the ARSA [7]. A high degree of suspicion for TEE-related compression should therefore be maintained, particularly in high-risk patients, such as neonates and low-birth-weight infants.

This case highlights the importance of recognizing vascular anomalies such as an ARSA in pediatric cardiac surgery. The mechanical compression of an ARSA by a TEE probe can result in misleading arterial line waveforms, complicating intraoperative hemodynamic assessment. A high index of suspicion, careful preoperative imaging review, and consideration of alternative monitoring sites are key to optimizing patient safety. Awareness of this phenomenon allows anesthesiologists and surgeons to troubleshoot arterial waveform abnormalities effectively, ensuring accurate monitoring and improved surgical outcomes.

### Learning points

ARSA is the most common vascular anomaly of a left-sided aortic arch and is frequently asymptomatic, which may lead to underrecognition on preoperative imaging. Because the ARSA typically follows a retroesophageal course, it is particularly susceptible to mechanical compression by a TEE probe. This case reinforces the prevalence of ARSA in patients with trisomy 21 and emphasizes the need for a high index of suspicion for vascular anomalies when unexplained intraoperative hemodynamic monitoring abnormalities occur. Furthermore, it emphasizes the importance of meticulous preoperative imaging review, as asymptomatic vascular anomalies may be overlooked when attention is primarily directed toward intracardiac defects.

## Data Availability

Any inquiries regarding supporting data availability of this study should be directed to the corresponding author.
